# Comparison of mixed model based approaches for correcting for population substructure with application to extreme phenotype sampling

**DOI:** 10.1186/s12864-022-08297-y

**Published:** 2022-02-04

**Authors:** Maryam Onifade, Marie-Hélène Roy-Gagnon, Marie-Élise Parent, Kelly M. Burkett

**Affiliations:** 1 Department of Mathematics and Statistics, University of Ottawa, Ottawa, Canada; 2 School of Epidemiology and Public Health, University of Ottawa, Ottawa, Canada; 3 Centre Armand-Frappier Santé Biotechnologie, Institut national de la recherche scientifique, Université du Québec, Laval, Canada

**Keywords:** Population stratification, Extreme phenotype sampling, Generalized linear mixed models, Type 1 error, Genome-wide association study

## Abstract

**Background:**

Mixed models are used to correct for confounding due to population stratification and hidden relatedness in genome-wide association studies. This class of models includes linear mixed models and generalized linear mixed models. Existing mixed model approaches to correct for population substructure have been previously investigated with both continuous and case-control response variables. However, they have not been investigated in the context of extreme phenotype sampling (EPS), where genetic covariates are only collected on samples having extreme response variable values. In this work, we compare the performance of existing binary trait mixed model approaches (GMMAT, LEAP and CARAT) on EPS data. Since linear mixed models are commonly used even with binary traits, we also evaluate the performance of a popular linear mixed model implementation (GEMMA).

**Results:**

We used simulation studies to estimate the type I error rate and power of all approaches assuming a population with substructure. Our simulation results show that for a common candidate variant, both LEAP and GMMAT control the type I error rate while CARAT’s rate remains inflated. We applied all methods to a real dataset from a Québec, Canada, case-control study that is known to have population substructure. We observe similar type I error control with the analysis on the Québec dataset. For rare variants, the false positive rate remains inflated even after correction with mixed model approaches. For methods that control the type I error rate, the estimated power is comparable.

**Conclusions:**

The methods compared in this study differ in their type I error control. Therefore, when data are from an EPS study, care should be taken to ensure that the models underlying the methodology are suitable to the sampling strategy and to the minor allele frequency of the candidate SNPs.

**Supplementary Information:**

The online version contains supplementary material available at (10.1186/s12864-022-08297-y).

## Background

In genetic studies involving human populations, researchers are interested in determining how genetic variation contributes to disease. Genome-Wide Association Studies (GWAS), which involve genotyping a large number of individuals at hundreds of thousands of genetic markers, have been useful for discovering relationships between common variants and complex diseases. Recently, sequencing has been used to discover rare variants associated with human traits [1]. Although the cost of genetic association studies has decreased over the years, some technologies, including sequencing, remain relatively expensive [2]. Therefore study designs that reduce cost while maintaining power are desirable.

An example of a cost saving design is extreme phenotype sampling (EPS), a design where genetic data are collected only on individuals in the tails of the phenotype distribution. The use of this study design was proposed by Lander and Botstein [3] for linkage analysis. Extreme phenotype sampling was later used for candidate gene association studies. For example, the EPS design was used to investigate associations between genetic variants in the dopamine system genes and cognitive ability [4, 5]. This study design has also been used in GWAS, for example in Vermissen et al. [6] to identify genetic risk variants for coronary heart disease. Recently, EPS has been shown to be a powerful design to detect rare variants [2, 7–9].

As with all population-based genetic association designs, extreme phenotype sampling is prone to confounding by population structure or stratification. Differences in allele frequencies among members of a strata or subgroup in the population may lead to confounding if there are also differences in the phenotype distribution between the subgroups. Confounding is known to inflate the type I error rate, which can lead to spurious associations. Methods have been developed that can correct for the effects of population stratification using genomic data. The earliest approaches include Genomic Control [10] and STRUCTURE/STRAT [11]. Principal components (PC)-based corrections have also been shown to be sufficient for controlling the false positive rate [12, 13].

Mixed model methods have recently become popular due to their robustness in tackling other sources of confounding in the study, in particular cryptic relatedness [14]. Since mixed model based approaches are computationally intensive, a number of exact and approximate linear mixed model (LMM) methods have been developed for use in genome-wide association studies (for example, [15–17]). Each of these methods incorporate different strategies to make the LMM-based analyses feasible at the genome-wide level. Eu-ahsunthornwattana et al. [18] gives a comparison of these methods.

In human genetic studies, the phenotype of interest is often a binary trait, such as presence or absence of disease. To correct for population stratification, binary traits are sometimes analysed using LMMs [19–21] even though the response variable is not continuous. Pirinen et al. [22] gives a justification of this approach by deriving a mapping between the effect size estimates from the linear to the log-odds scale, which is the natural scale for binary traits. Although widely applied to binary traits, the LMM assumes a continuous phenotype with a constant residual variance. However, for binary traits in the presence of covariates, this assumption does not hold. Therefore, fitting a binary response with linear mixed models may fail to correct the type I error rate [23] or result in a loss of power [24].

Mixed model approaches that do not treat disease status as a continuous random variable have recently been developed. One such approach is based on the liability threshold model, which assumes that there is an unobserved normally distributed latent variable known as the ‘liability’ and that individuals having liability values above a threshold are classified as cases. Liability threshold-based methods have been implemented in the software LEAP [25] and LTMLM [26]. These methods estimate the latent liabilities and association is tested using these estimated latent response values. The generalized linear mixed model (GLMM) can also be used to model binary traits. For example, GMMAT [23] fits a logistic mixed model to the binary data, while CARAT [27] fits a retrospective model using a quasi-likelihood approach.

We have previously shown that the false positive rates due to population stratification are substantially inflated with EPS designs relative to random sampling [28]. Therefore, for EPS designs it is very important to include correction for population stratification. We have shown that including the top principal components in a logistic regression model adequately limits the type I error rate when the candidate variant was common; however, there was a slight inflation when the candidate variant was rare [28]. The mixed model-based approaches for correcting for population substructure were developed assuming binary traits from case-control type studies. In particular, the retrospective and liability threshold approaches model the underlying case-control ascertainment. However, the sampling scheme used in EPS designs is different from true case-control designs as both extremes of the phenotype distribution are included. Therefore, it is unclear whether these approaches will adequately control the false positive rate under the EPS ascertainment scheme when there is confounding due to population stratification. Given the increasing popularity of mixed model approaches, it is important to assess their performance in the EPS setting.

In this work, we aim to accomplish two goals. First, we present an overview of the mixed model-based approaches for correcting for population stratification with a binary response variable; we focus on the recently proposed algorithms LEAP, LTMLM, GMMAT and CARAT. Second, we compare the performance of these approaches and an LMM approach (GEMMA [17]) when the binary data comes from an EPS design. We use simulation to evaluate whether the type I error rate is adequately controlled when the candidate variant is both common and rare. We also examine the power of the methods shown to control the type I error rate. Finally, we compare these methods when applied to a real dataset collected as part of a case-control study conducted in Québec, Canada. The participants were collected from multiple ethnic groups and therefore we expect confounding by population stratification with this data.

## Results

### Evaluation of type I error - common variant

Table 1 shows the estimated type I error rates for the EPS samples of size 1000, 2000 and 4000, which correspond to full cohort sample sizes of 5000, 10,000 and 20,000 individuals. These results correspond to the simulations with the ‘1’ allele frequency of *p*_1_=0.25 and *p*_2_=0.85 and the phenotypic means of *μ*_1_=0.07 and *μ*_2_=−0.07 for subpopulations 1 and 2, respectively. LEAP and GMMAT show well controlled type I error rates, indicating adequate correction of the population structure in the data. For both approaches, the estimated type I error rate for all the sample sizes ranges between 0.041−0.052. All but one of these estimates are slightly lower than the nominal level of 0.05; however, these small deviations from the true value can be explained by Monte Carlo sampling error. The type I error rate for the PCA approach is also close to the nominal value, though possibly slightly elevated; similar results for the PCA based correction were observed in our previous work [28]. CARAT shows higher type I error rates than the nominal level of 0.05. The false positive proportion ranges from 0.089 to 0.102, which is higher than can be explained by Monte Carlo simulation error alone. We therefore conclude that CARAT is not able to adequately correct for population stratification in the EPS setting.

**Table 1:** Estimated type I error rates for the three mixed model approaches for binary traits (LEAP, GMMAT and CARAT), the LMM method (GEMMA) and logistic regression with principal component based correction (PCA)

Cohort Sample Size (N)	Sub-sample Size (0.2N)	LEAP	GMMAT	CARAT	GEMMA	PCA
5000	1000	0.0405	0.04135	0.102.	0.061	0.0575
10000	2000	0.0417	0.0475	0.089	0.046	0.0605
20000	4000	0.0450	0.0515	0.0945^∗^	0.052	0.0555

*Based on *m*=1999 simulations

We also evaluated the LMM approach GEMMA, where we coded the categorical phenotype as 0 and 1 for the two extreme groups and treated the 0/1 values as a continuous phenotype. Results in Table 1 show that the estimated type I error rates were around 0.05, which indicates that erroneously analysing as a continuous trait does not affect the correction for population substructure.

**Fig. 1:**
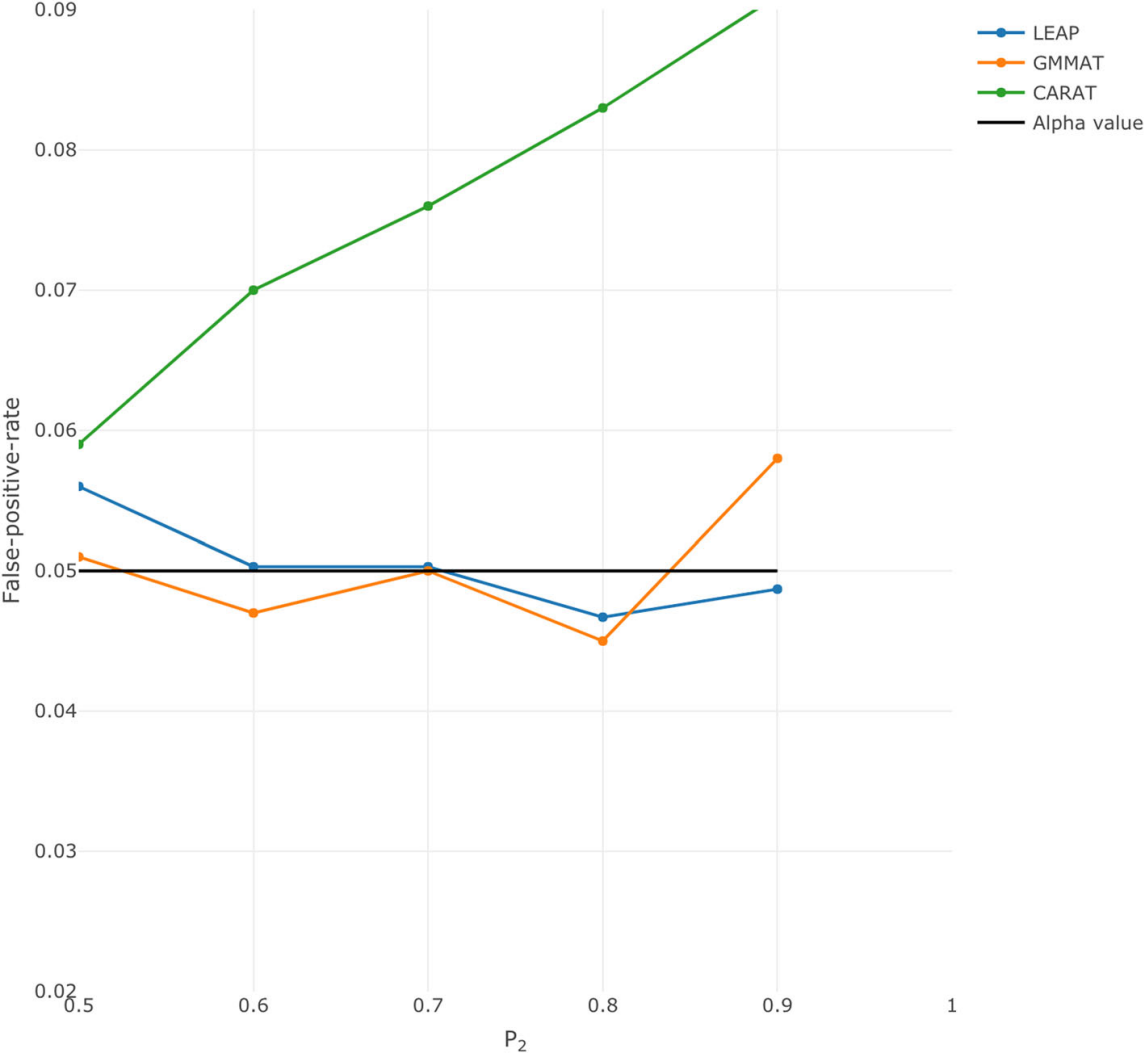
Type 1 error rates for the three mixed model methods (LEAP, GMMAT and CARAT). The allele frequency in population 1, *p*_1_, was fixed at 0.5. The allele frequency in population 2, *p*_2_, ranged from 0.5 to 0.9. The x-axis corresponds to the *p*_2_ value. The orange line represents GMMAT, the blue line represents LEAP, and the green line represents CARAT. The horizontal line indicates the alpha value of 0.05

Figure 1 shows the results when the ‘1’ allele frequency of the candidate SNP in subpopulation 2 was varied from 0.5 to 0.9, in increments of 0.1. When *p*_1_=*p*_2_ there is no population stratification; as expected, under this case the type I error rate of the three methods are all close to the nominal value of 0.05. GMMAT and LEAP show no increase in the estimated type I error rates as *p*_2_ increases; the estimated value remains around 0.05. However, for CARAT the type I error rates increases as the difference in the allele frequency between the two subpopulations increases, which again indicates inadequate correction for population stratification.

### Evaluation of type I error - rare variants

**Fig. 2:**
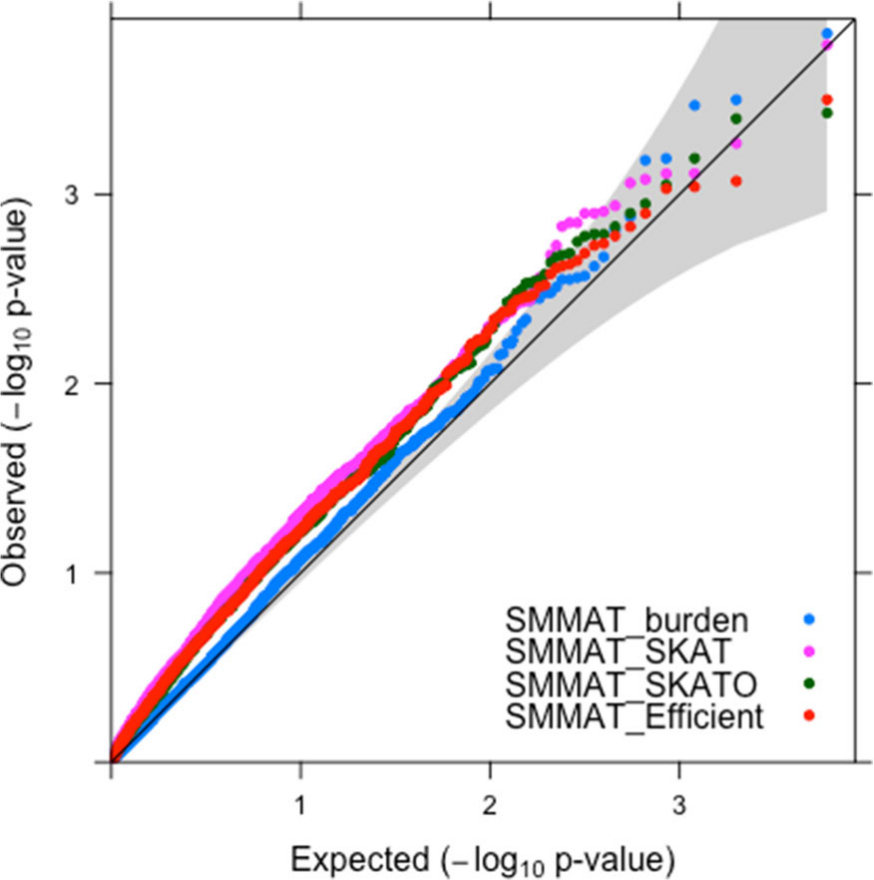
Quantile-Quantile Plots of the − log10 of the *p*-values from the four SMMAT rare variant association tests (Burden, SKAT, SKAT-0 and Efficient)

Table 2 shows the estimated type I error rates of the Burden (SMMAT-B), SKAT (SMMAT-S), SKAT-O (SMMAT-O) and Hybrid efficient (SMMAT-E) statistics from SMMAT assuming a significance level of 0.05. The Burden test had an estimated type I error rate closest to the specified value (0.062 versus the expected 0.05). The three other rare variant statistics (SMMAT-S, SMMAT-O, SMMAT-E) have estimated type I error rates that range from 0.088 to 0.103. The inflation of the test statistics can also be seen in the QQ plot of the -log10 of the *p*-values (Fig. 2); the results with the Burden statistic appear closest to the identity line, which is what we would expect under no association, but there is still evidence of inflated test statistics. The deviations between the true and estimated type I error rates cannot be explained by simulation error alone; we conclude that under EPS, the type I error rate is not controlled using these rare variant tests.

**Table 2:** Estimated type I error rates for the rare variant mixed model methods implemented in SMMAT

SMMAT Method	Estimated Type I Error Rate
Burden (SMMAT-B)	0.0617
SKAT (SMMAT-S)	0.1039
SKAT-O (SMMAT-O)	0.1024
Efficient (SMMAT-E)	0.0883

### Evaluation of power

Table 3 shows the estimated power to detect a common candidate variant when the phenotype also depends on subpopulation membership. We assessed power only for the methods with appropriate type I error control and we evaluated two effect sizes. We note that overall power for all methods will depend on the effect size and sample size; therefore, we focus on comparing the estimates from each method to each other rather than on determining if power overall is high enough. At the larger effect size (*β*=0.25), no method clearly outperforms the others. The estimated power for all four methods ranges from 0.48 to 0.52. LEAP has the lowest power and GEMMA the highest. The same pattern is seen with the lower effect size (*β*=0.15); LEAP is lowest and GEMMA is highest. At the smaller effect size, GEMMA’s estimated power is about 10% higher than the next lowest (PCA). If we perform a test of equality of proportions estimated for GEMMA and PCA, we would reject the hypothesis that they are equal.

**Table 3:** Estimated power for detecting a causal variant of two different effect sizes (*β*=0.15 and *β*=0.25) in the presence of population stratification

Method	Estimated Power	
	*β*=0.15	*β*=0.25
LEAP	0.31	0.48
GMMAT	0.34	0.51
GEMMA	0.44	0.52
PCA	0.35	0.49

### Extreme BMI phenotype in the prostate cancer case-control study

**Fig. 3:**
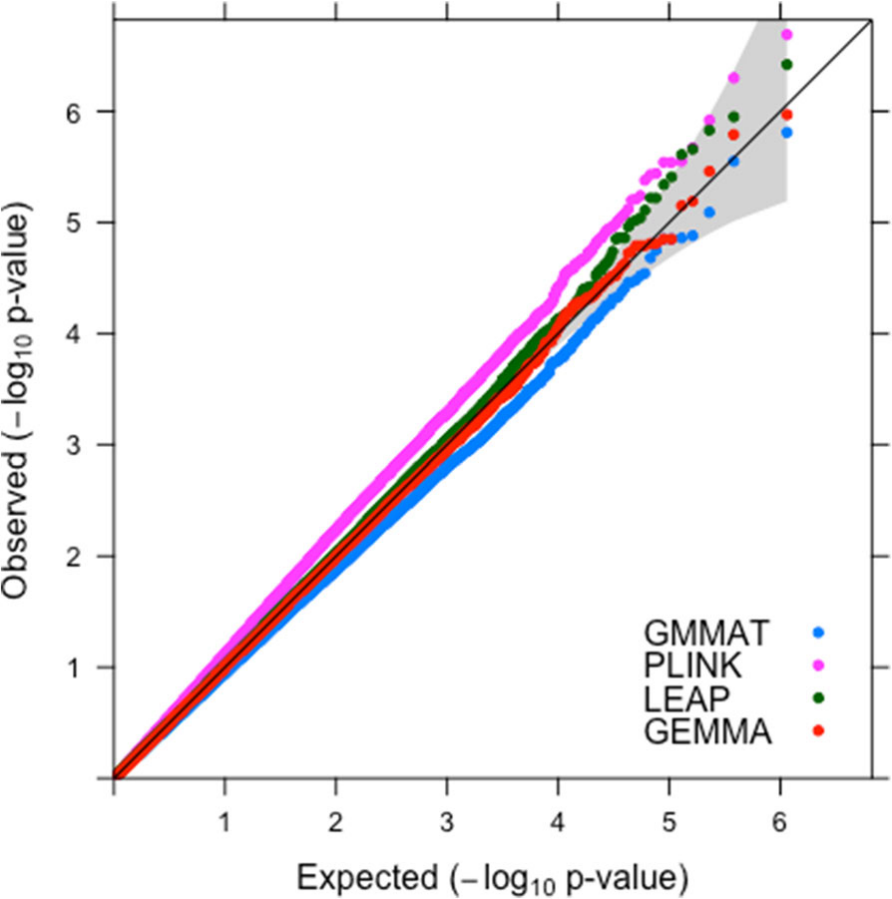
Quantile-Quantile plot of population stratification adjusted GMMAT, LEAP, GEMMA and uncorrected PLINK in the GWAS analysis of the case-control dataset

Figure 3 shows the QQ plots of − log10 of the *p*-values from LEAP, GMMAT, GEMMA and the uncorrected logistic regression implemented in PLINK for the genome-wide association study using the extremes of the BMI phenotype from the prostate cancer case-control study. For reference purposes, Manhattan plots for each method are provided in Supplementary Figures 1-4 (Additional files 1, 2, 3 and 4), respectively.

The results from LEAP, GEMMA, and GMMAT show well controlled type 1 error rates; in Fig. 3, the majority of *p*-values tend to fall close to the identity line although again GMMAT may slightly over-correct. The correction for relatedness does seem to alter the results; we can see that the points for the methods that offer correction (GMMAT, GEMMA and LEAP) are all below the points for the method which doesn’t correct (PLINK).

### Computational time and memory usage

We compared the computational time and memory requirements for LEAP, GMMAT, GEMMA and CARAT on a dataset of *n*=1000 individuals. With the exception of GMMAT, the methods included GRM calculation as part of the algorithm. For GMMAT, we used the GRM computed by GEMMA; this extra step increases the overall computational time. The average time (across simulations) to complete an association test on a single candidate SNP was approximately 22 seconds for GMMAT, 19 seconds for GEMMA, and 13 seconds for LEAP. Although the times are similar when analysing a single SNP, these differences between run times would be quite noticeable for a GWAS analysis. CARAT’s run time was significantly longer at over 5 minutes per dataset; we therefore were unable to complete the same number of simulations for CARAT at the larger sample size (4000 samples from the phenotype extremes). Memory usage was comparable between methods, though LEAP’s usage was higher (GMMAT: 3 GB, GEMMA: 2 GB; LEAP: 4.25 GB; CARAT 1.34 GB).

## Discussion

In this work, we compared the performance under an EPS design of several mixed model-based association methods for binary phenotypes. We estimated the type I error rate for all methods under both a common variant and a rare variant scenario. We evaluated power for those methods with appropriate type I error control and we compared the computational requirements of the methods. We also applied the methods to a real dataset that was known to have population substructure.

For common variants, our simulations showed that methods based on the generalized linear mixed model (GMMAT), the LMM (GEMMA) and the liability threshold model in conjunction with an LMM (LEAP) all have a type 1 error rate that is close to, or at least not higher than, the specified value. Although, Chen et al. [23] note that the liability threshold mixed models may fail to control the type 1 error rates in the presence of moderate to strong population stratification, we did not observe such inflation in our simulations even when confounding due to population stratification would have been severe. On the other hand, we found that CARAT, which uses a retrospective model and a quasi-likelihood framework, did not adequately control the type 1 error rate. The CARAT method is based on a retrospective approach where the case-control ascertainment is modeled [24]. Though this is an appropriate approach for a true case-control design, extreme sampling represents a different type of ascertainment and therefore the retrospective model may not be appropriate in this case.

For rare variants, the type 1 error rate was inflated relative to the specified level for all statistics implemented in the GLMM-based approach SMMAT. The burden test had type 1 error closest to the nominal value of 0.05, which may be explained by the lower power of the burden test overall relative to the optimized variance component tests like SKAT-O [29] (SMMAT-O). Under population stratification there is a true difference between the genotype distributions in the two extreme groups, though this difference is not due to a causal association between the genetic variant and the phenotype. Therefore, methods that have higher power overall, like SKAT-O, will be more likely to detect this false association. Studies have shown that the inflation due to population stratification is higher with rare variants than with common variants (for example, [30]). Using a SKAT-type method incorporating a mixed model-based correction with common variants has been shown to adequately control the type 1 error under random sampling [31]. However, EPS inflates the effects of population stratification to a greater extent than random sampling [28]; therefore, conclusions drawn about corrections with rare variant approaches under random sampling scenarios may not apply to the EPS setting. In addition, the SNPs we simulated for the GRM calculations were all common. It is possible that in the context of EPS, a mixture of rare and common variants for capturing ancestry might have better type 1 error control since it was shown to be slightly conservative in the random sampling setting [31].

We also investigated the performance of LEAP, GMMAT and GEMMA for detecting genetic variants associated with the extremes of BMI in the prostate cancer case-control study. Although we do not know whether there are true associations in this dataset, we note that LEAP, GMMAT and GEMMA all have different genome-wide *p*-value distributions than the uncorrected results (logistic regression with PLINK), and that the corrected distributions appear to have less overall inflation of the test statistics. However, the results for GMMAT indicate a slight over-correction. In our common candidate variant simulations, we also observe some over-correction with both GMMAT and LEAP at the smaller sample sizes. Therefore, it is possible that the over-correction can be explained by the small sample size of the BMI EPS dataset.

The use of LMMs for binary traits has been discouraged due to the fact that this approach ignores the meanvariance relationship of the binary model and instead assumes a constant relationship [23]. Chen et al. [23] demonstrate both an increase and decrease in false positives with an LMM approach on a stratified asthma dataset by separating cases where the variance of the MAF was higher/lower in one ethnic group relative to remaining groups. In our simulations, GEMMA’s LMM approach did not have an inflated false positive rate even under moderate to strong population stratification; though we note that our simulations were not designed to investigate this thoroughly. For example, in our simulations we did not vary the proportion of the full cohort from each subpopulation. In addition, in the real data analysis of the BMI phenotype, the results from GEMMA were actually closest to what would be expected if there were no true associations. Therefore, for both the simulated and real data, GEMMA had very good correction of the false positive rate when compared to the other methods.

A weakness of our simulation is the use of the Balding Nichol’s model in simulating genotype data for GRM estimation. The Balding’s Nichols’s model allows the allele frequencies to differ between the subpopulations and guarantees a specific *F*_*st*_ value. However, for a given SNP, the actual allele frequency difference between the two subpopulations is small. In real data, some SNPs are highly differentiated between subpopulations [32]; these types of SNPs would not be simulated under this model.

In this study, we model the extreme phenotypes as binary and use methods suitable for analysing case-control or binary data. However, Barnett et al. [33] point out that analysing extremes as a binary phenotype rather than using the quantitative values might lead to a reduction in power to detect genotype-phenotype associations. However, if using the quantitative phenotype values, the extreme sampling mechanism must be modeled. For example, Lin et al. [34] showed that parameter estimates from the linear model are biased when the quantitative phenotypes are naively analysed without accounting for the sampling. Linear model-based methods that model the quantitative phenotype while accounting for the extreme sampling scheme have been developed [34, 35]. However, no such approach currently exists for the linear mixed model; this is therefore a topic for further research.

## Conclusions

The mixed model-based methods for population stratification correction compared in this study do not all perform equally well when the data is taken from an extreme sampling design. For common variants, LEAP, GMMAT and GEMMA all had good type I error rates and power; however, CARAT did not adequately control the type I error rate. In addition, none of the available mixed model approaches for rare variants controlled the type 1 error rate. Therefore, when the data are from an EPS study, care should be taken to ensure that the underlying models used in the methods are suitable to the sampling strategy and to the minor allele frequency of the candidate SNPs. Our study highlights the need for the development of mixed model-based approaches for population stratification correction that model the underlying sampling structure of the EPS design and are applicable to variants of all frequencies.

## Methods

### Overview of mixed model-based approaches for correcting for population stratification

In this section, we give a brief overview of mixed models and implementations that incorporate these models to correct for population structure. We focus on approaches that are suitable for binary response variables.

#### The linear mixed model and the generalized linear mixed model

A linear mixed model (LMM) to account for population substructure and/or hidden relatedness is given by:

1 $$  Y = X\beta + Zb + \epsilon  $$

where *Y* is the vector of phenotype values, *X* is the design matrix of genetic and non-genetic fixed-effect covariates including a column vector of 1, *β* is the vector of regression coefficients including the intercept, *Z* is a known design matrix corresponding to clustering that is the identity matrix in the simplest case and *b* is the vector of random effects. We assume the random effects, *b*, are $N(0, \sigma _{a}^{2}G)$ distributed, where *G* is the known relationship matrix and $\sigma _{a}^{2}$ is the additive genetic variance, and $\epsilon \sim N(0,\sigma ^{2}_{e}I)$, where $\sigma ^{2}_{e}$ is the error variance and *I* is the identity matrix. Therefore, the distribution of *Y* is:

2 $$  Y \sim \mathcal{N}\left(X\beta + Zb, \sigma_{a}^{2} G + \sigma_{e}^{2} I\right)  $$

We can infer from (2) that the matrix *G* imposes structure on the covariance matrix of *Y*; this forms the basis of using LMMs to correct for hidden relatedness in GWAS. With population-based samples, the relationship matrix *G* is estimated using genome-wide data.

Model (2) can be generalized to handle non-normal response variables. Given a vector of random effects *b*, the response variable *Y* is assumed to be from a distribution in the exponential family. That is, for the *ith* response,

$$f_{i}(y_{i} | b) = \exp \left\lbrace\frac{y_{i} \varphi - b^{*}(\varphi)}{a_{i}(\phi)} + c_{i} (y_{i}, \phi)\right\rbrace $$

where *b*^∗^(.),*a*_*i*_(.),*c*_*i*_(.,.) are known functions that depend on the underlying distribution of *Y*, *φ* is a parameter that is associated with the conditional mean *μ*_*i*_=*E*(*Y*_*i*_|*b*), and *ϕ* is a dispersion parameter which may or may not be known. The linear predictor is *η*_*i*_=*x*_*i*_*β*+*z*_*i*_*b*, where *x*_*i*_ and *z*_*i*_ are the covariates for the *i*th individual and *β* and *b* are as previously defined. The mean for individual *i*, *μ*_*i*_, is related to the linear predictor via a link function:

$$g(\mu_{i}) = \eta_{i}.$$

In particular, the mixed logistic model for a binary response variable is given by

3 $$ \text{logit}(p_{i}) = x_{i}\beta + z_{i} b,  $$

where *p*_*i*_= Pr(*Y*_*i*_=1|*b*) and *x*_*i*_,*z*_*i*_ and *b* are as defined above.

#### Summary of mixed model implementations

Recently, several mixed model approaches for binary traits have been developed. In this section, we summarize the different approaches that have been implemented, which we classify as (i) approaches using the LMM, (ii) approaches using liability threshold models in conjunction with the LMM, and (iii) GLMM-based approaches. We provide more detail on the liability threshold (ii) and GLMM (iii) approaches since the LMM implementations (i) have been compared and summarized elsewhere [18, 36].

*(i) Linear Mixed Model approaches*

As previously mentioned, LMMs are used with binary traits even though the response variable is neither normal nor continuous. In order to fit LMMs in the GWAS context, large sample sizes are required to achieve sufficient statistical power. Unfortunately, the computational complexity associated with fitting LMMs increases cubicly with the number of individuals in the model [37]. This motivated the development of several variations of the LMM approach designed to increase computational speed and in turn make large scale GWAS feasible. Existing methods include EMMA [15], EMMAX [38], FASTLMM [16], BOLT-LMM [39, 40], GCTA [41], and GEMMA [17]. Some of these approaches have been designed to handle some specific forms of binary data. For example, BOLT-LMM is able to analyse balanced case-control data at large sample sizes [40].

*(ii) Liability threshold models in conjunction with the LMM*

In case-control studies, cases are over-sampled relative to the disease prevalence. The liability threshold model (LTM) assumes an underlying but unobserved latent trait that is normally distributed [42, 43]. Individuals with latent trait values beyond a threshold, *t*, are classified as cases (*Y*=1) and all others are classified as controls (*Y*=0). Hence the binary response variable for individual *i*, can be written as:

4 $$ Y_{i} = \left\{\begin{array}{ll} 1 & \; \text{if} \; z_{i} > t \\ 0 & \; \text{otherwise} \end{array}\right.  $$

where *Y*_*i*_ is the observed binary trait and *z*_*i*_ is the unobserved liability score, which is assumed to be *N*(0,1). Since the liability scores are not observed, using the liability threshold model requires first estimating liability scores for each individual. We now describe two implementations which differ in how the liability scores are estimated.

In the algorithm LEAP [25], the liability for individual *i* is assumed to be a sum of genetic and environmental components, *z*_*i*_=*g*_*i*_+*e*_*i*_, where $g_{i} = X_{i}^{t}\beta _{g}, X_{i}^{t}$ is the vector of genotype data and $e_{i} \sim N(0, \sigma ^{2}_{e})$. Estimation of *z*_*i*_ is achieved by first fitting a regularized probit model to estimate the parameters *β*_*g*_. These are estimated with the maximum a posteriori estimate (MAP), also known as the posterior mode estimator. The liabilities are estimated by $\hat {z_{i}} = X_{i}^{t}\hat \beta _{g} $; these values are then used as the phenotype values for each individual. Tests for association are performed using a linear mixed model since the liabilities are assumed to be normally distributed.

LTMLM [26] is similar to LEAP in that it models the retrospective sampling and uses imputed liability scores; however, the liabilities are estimated using the posterior mean of the multivariate liability distribution (PMLs). A Gibbs sampler is used to sample from this distribution and the posterior mean is estimated by averaging over the Monte Carlo iterations. A score statistic is used to test for association between a candidate SNP and the imputed liabilities assuming a linear mixed model.

A comparison of the estimators used by LEAP and LTMLM showed that in the presence of population structure, the MAP yields more accurate liability estimates than the PML, often at a lower computational cost compared to the posterior mean estimator [25].

*(iii) GLMM-based approaches*

The logistic mixed model is a special case of the GLMM that can be used to analyse binary traits while accounting for population structure and hidden relatedness. However, this model has not been widely used for GWAS due to the computational complexity involved in fitting logistic mixed models for a large number of genetic variants. Chen et al. [23] developed GMMAT, a logistic mixed model that is computationally efficient enough to handle genome-wide data. GMMAT first fits a null logistic mixed model including fixed effects for any covariates and random effects for residual population stratification and relatedness. This fitted null model, which is the same for all genetic variants in the study, is then used to test for the association between a genetic variant and phenotype using a score test. The use of just one null model for testing all genetic variants greatly simplifies the model compared to fitting a full logistic mixed model for a large GWAS.

CARAT (Case control Retrospective Association Test) [27] is another mixed model approach for binary traits where the response variable is modeled using a mixed effects quasi-likelihood approach. In particular, only the conditional mean and covariance of the response variable given the genotypes and other covariates are specified. The conditional mean is selected to be the same as for the logistic model. The conditional covariance incorporates features of the logistic model and accounts for population substructure through the genetic relationship matrix. Like LTMLM, CARAT uses a retrospective model where the genotypes are treated as random and the association is performed conditional on the phenotypes and non-genetic covariates. However, unlike LTMLM, CARAT does not require the knowledge of disease prevalence. Like LTMLM, a score test is used to handle genome-wide data.

### Simulations to evaluate type I error

In this section, we describe the simulation studies used to estimate the type I error rates of the mixed model software implementations that handle binary data. In particular, we focus on LEAP, GMMAT, CARAT and GEMMA as a representative LMM approach. We excluded LTMLM as we found that it took much longer to run than LEAP, which uses a similar liability threshold model.

#### Common candidate variant

We assumed a cohort consisting of two subpopulations of equal proportion. The total cohort size, *N*, was set to 5000,10,000 or 20,000. The *F*_*st*_ value between the two populations - a measure of genetic population differentiation - was set to 0.01; this value is higher than would be expected between typical European populations but it ensures substantial substructure [28].

Genetic data was simulated using the Balding-Nichols method [14, 44] as previously described [28]. For each individual, we simulated a total of *p*=5000 bi-allelic SNPs. Though true genome-wide data would consist of much larger numbers of SNPs, our previous work with data simulated using this model has shown that this number of SNPs is sufficient to correct for population stratification [28]. We label the two alleles at each SNP as either ‘0’ or ‘1’. For each SNP, the generating allele frequency, *p*, for the ‘1’ allele was first sampled from a uniform [0.1,0.9] distribution. To mimic population differentiation, the ‘1’ allele frequency within each of the two populations, *p*_1_ and *p*_2_, was then sampled from a Beta distribution with shape and scale parameters $\frac {p(1-F_{st})}{F_{st}}$ and $\frac {(1-p) (1-F_{st})}{F_{st}}$, respectively. This approach has been shown to generate genotype data having the desired *F*_*st*_ level [44]. Using the allele frequencies generated for each population, the genotype data was sampled assuming Hardy Weinberg equilibrium. The genotype data was coded as 0, 1 or 2 for genotypes 00, 01, and 11, respectively.

We simulated a candidate SNP separately. We first assumed that the ‘1’ allele frequency for the candidate SNP was *p*_1_=0.25 in the first subpopulation and *p*_2_=0.85 in the second subpopulation. Although this allele frequency difference is probably not realistic in practice, it was chosen to reflect a ‘worst case’ scenario of a candidate SNP showing extreme population differentiation. We also included a smaller simulation where we varied the ‘1’ allele frequency difference between the populations; in particular, we set *p*_2_ to range from 0.5−0.9 while fixing *p*_1_ at 0.5.

In order to obtain the EPS sample, we simulated phenotypes from a normal distribution with mean values *μ*_1_=0.07 and *μ*_2_=−0.07 for subpopulation 1 and 2, respectively, and a common variance of *σ*^2^=1. We note here that the genotypes and phenotypes have been simulated independently, which implies that the genotype at the candidate SNP is not causally associated with the phenotype. The EPS sample was then selected as the individuals in the upper and lower 10*th* percentile of the phenotype distribution. For the EPS sample, the binary response variable is membership in the upper or lower group; in practice, these are sometimes labelled as cases and controls, though it should be noted that there is no true control group in this design.

For methods requiring a GRM, the genetic data on the *p*=5000 SNPs simulated under the Balding-Nichols method was used to compute the GRM; the candidate SNP for the association test was not included in the GRM calculation. LEAP and GEMMA compute the GRM as part of the algorithm. For GMMAT, the GRM must be computed externally and then passed to the program; we used the standardized GRM computed by GEMMA.

We simulated *m*=3000 datasets under the scenario where the candidate variant ‘1’ allele frequency was *p*_1_=0.25 and *p*_2_=0.85 in subpopulation 1 and 2, respectively. The computational time for CARAT is significantly longer than the other methods, particularly for the large sample sizes. Therefore, we were only able to complete CARAT analysis of *m*=1999 simulated datasets for the simulation with full cohort size of *N*=10,000. Due to limited computational time, we only performed *m*=1000 simulations for each setting under the scenario where *p*_1_=0.5 and *p*_2_ varied. For these simulations, we chose to focus on the trend in the rate as *p*_2_ varied for each method separately.

GMMAT is available as an R package [23]. LEAP, GEMMA and CARAT are stand-alone software packages that can be run at the command line on a Unix operating system. We used default settings for all packages. For comparison purposes, we also included a PC-based correction by including the top 5 principal components in a logistic regression model; this was also done in R. For each method, the type I error rate was estimated by the proportion of the simulated datasets where the null hypothesis was rejected at level *α*=0.05. Simulations were run in a cluster computing environment (CAC-FRONTENAC) and all analysis of the results was done in R [45].

#### Rare candidate variants

We also investigated the performance with a candidate region having rare variants. To simulate data for the candidate region, we simulated haplotype data in a 30kb region using the coalescent-based simulation program *ms* [46]. We simulated a total of 10,000 haplotypes assuming an effective population size of *N*_*e*_=100,000, a per-site mutation rate of *μ*=10^−8^ and a per-adjacent site recombination rate of *ρ*=10^−8^. To incorporate population structure, we again assumed two subpopulations of equal size (i.e. 5000 haplotypes from each subpopulation) and a migration parameter *M*=10, which is representative of the population differentiation parameter *F*_*st*_=0.01 in the case of a common variant [30]. To create genotypes in the candidate region for *N*=5,000 individuals, the 10,000 haplotypes were randomly paired within subpopulation. The continuous phenotype values and genetic data at 5000 non-candidate SNPs (for GRM estimation) were generated as previously described for the common variant simulation study.

To test for association with rare variants while accounting for population structure, only the generalized linear model approach had software available. We used SMMAT (variant set mixed model association test) [47], which is a function available in the GMMAT package to perform several popular rare variant tests (burden test [48], SKAT [1], SKAT-O [49], and an efficient hybrid test that combines the burden and SKAT tests [47]) in the binary mixed model framework. We used the default values set in the software for all tests. As with the common variant scenarios, we estimated the type I error by the proportion of tests rejected at level *α*=0.05.

### Simulation to investigate power

For methods that adequately controlled the type I error rate (LEAP, GMMAT, GEMMA, PCA), we conducted additional simulations in order to compare their performance with respect to power. We did not include a rare variant power simulation since none of the methods we tested adequately controlled the type I error rate.

For the power simulations, the genetic data for estimating ancestry was simulated using the same procedure as described for the type I error simulations. In particular, we continue to assume that there is hidden population subdivision. To simulate the candidate SNP, we assumed no differences in allele frequency between the two populations and an allele frequency of 0.2 for the causal allele. The genotypes were sampled assuming Hardy-Weinberg equilibrium. The phenotype was again simulated assuming a normal distribution, with variance 1 and mean *μ*_*i*_+*βG*_*ij*_ where *μ*_*i*_, the subpopulation means, are the same as for the type I error simulations, *G*_*ij*_ is the genotype of individual *j* in subpopulation *i*, and *β* is the effect size of the causal allele (*β*=0.15 and *β*=0.25). We simulated a full cohort size of *N*=10,000 which gives us an EPS subsample of *n*=2000 when we select the top and bottom 10%. The number of simulations for power estimation was set to *m*=3000 and power was estimated by the proportion of tests rejected at level *α*=0.05.

### Analysis of BMI phenotype from a prostate cancer case-control study

We evaluated the mixed model methods for common variants on data collected from a population-based case-control study, conducted in Montréal, Canada. The study has been described elsewhere (e.g. [50]). Briefly, cases were men aged 76 and under who were newly diagnosed with prostate cancer between 2005-2009; age-matched controls (in 5 year age groups) were randomly recruited from the electoral list of men in the same districts as cases. Overall, 1933 cases and 1994 controls were recruited into the study. Genome-wide genotyping was done using the Illumina OmniExpress 12 platform. We performed quality control which included removing SNPs and individuals with a missingness level above 0.02, minor allele frequency (MAF) below 0.05 and those that deviated from the Hardy-Weinberg equilibrium at a *p*-value of 10^−6^. We also checked that all the SNPs used were autosomal (i.e on chromosomes 1-22) and that all reported male individuals had an F value (based on the X chromosome inbreeding estimate) above 0.8. After quality control, genotype data was available on 574,885 SNPs and for 1295 cases and 1248 controls.

Data was collected on several continuous variables within this study. We found that body mass index (BMI) was not associated with prostate cancer status in this study (*P*-value=0.48); we therefore used this as our continuous phenotype and pooled the cases and controls. We selected those in the top and bottom 15% of BMI in our extreme sampling design. After data cleaning, we observed that 2520 of the men with complete genotype data also had BMI data. With these numbers, the sample size of the final EPS sample was about 756.

The study includes men from different ethnic backgrounds. About 77 of the men were Black, 28 were Asian, 1199 were European and 71 were of other nationalities. The ethnicity of 14 of the total sample collected could not be ascertained and therefore was marked as missing. As we are interested in methods for correcting for population stratification, we did not stratify our analysis by ethnicity.

We performed a GWAS comparing the methods GMMAT, LEAP and GEMMA. We excluded CARAT since we found that it had poor false positive rate correction in our simulations. Since this is a real dataset, we do not know whether there are true associations and whether population stratification is truly a problem. For this reason, we also used PLINK [51] to assess genome-wide association with no population stratification correction as a baseline comparison. For each method, we compute *p*-values of association for all available SNPs. We summarize the association results with Manhattan plots and we use QQ-plots of − log10 of the *p*-values to visually assess the inflation of test statistics. Both plots were created using the qqman R package [52].

## Supplementary Information

**Additional file 1** Title: Supplementary Figure 1. Description: Manhattan plot for results obtained from LEAP for the GWAS with the BMI phenotype. The y-axis shows -log10 of the *p*-values from the test for association between BMI extremes and genotype and the x-axis shows genomic position of the SNP. The blue line indicates the standard threshold for a suggestive association (*p*-value <1×10^−5^). The red line indicates the standard threshold for a genome-wide significant association (*p*-value <5×10^−8^).

**Additional file 2** Title: Supplementary Figure 2. Description: Manhattan plot for results obtained from GMMAT for the GWAS with the BMI phenotype. The y-axis shows -log10 of the *p*-values from test for association between BMI extremes and genotype and the x-axis shows genomic position of the SNP. The blue line indicates the standard threshold for a suggestive association (*p*-value <1×10^−5^). The red line indicates the standard threshold for a genome-wide significant association (*p*-value <5×10^−8^).

**Additional file 3** Title: Supplementary Figure 3. Description: Manhattan plot for results obtained from GEMMA for the GWAS with the BMI phenotype. The y-axis shows -log10 of the *p*-values from test for association between BMI extremes and genotype and the x-axis shows genomic position of the SNP. The blue line indicates the standard threshold for a suggestive association (*p*-value <1×10^−5^). The red line indicates the standard threshold for a genome-wide significant association (*p*-value <5×10^−8^).

**Additional file 4** Title: Supplementary Figure 4. Description: Manhattan plot for results obtained from PLINK (uncorrected logistic regression) for the GWAS with the BMI phenotype. The y-axis shows -log10 of the *p*-values from test for association between BMI extremes and genotype and the x-axis shows genomic position of the SNP. The blue line indicates the standard threshold for a suggestive association (*p*-value <1×10^−5^). The red line indicates the standard threshold for a genome-wide significant association (*p*-value <5×10^−8^).

## Data Availability

The computational scripts used for the simulation studies are available at https://github.com/statgen-uottawa/Onifade-EPS-PopStrat-GLMM. The prostate cancer case-control dataset that support the findings of this study are available from Dr. Parent but restrictions apply to the availability of these data, which were used under license for the current study, and so are not publicly available.

## Abbreviations

EPS: Extreme Phenotype Sampling
GWAS: Genome-Wide Assocation Study
SNP: Single Nucleotide Polymorphism
PC/PCA: Principal Components/Principal Component Analysis
LMM: Linear Mixed Model
GLMM: Generalized Linear Mixed Model
LTM: Liability Threshold Model
MAP: Maximum a posteriori estimate
PML: Posterior mean of the Multivariate Liability
MCMC: Markov chain Monte Carlo
GRM: Genetic Relationship Matrix
BMI: Body Mass Index
MAF: Minor Allele Frequency
