# Supplementary figures and images for: Comparison of mixed model based approaches for correcting for population substructure with application to extreme phenotype sampling
Source: BMC Genomics. 2022 Feb 4;23:98. doi: 10.1186/s12864-022-08297-y (PMC8815214; doi:10.1186/s12864-022-08297-y)

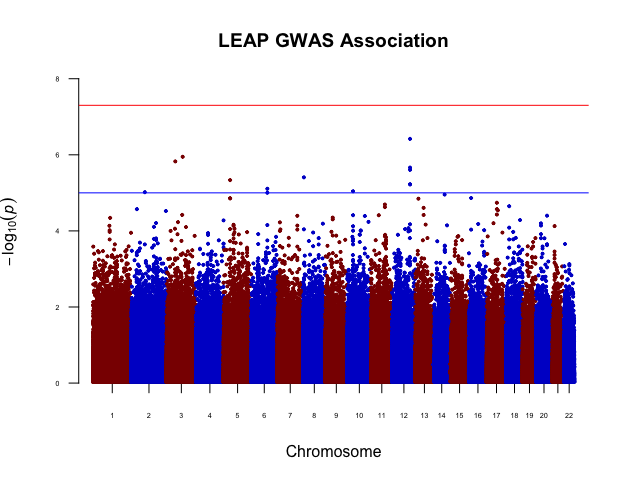

Supplement: Supplementary file 1 — Additional file 1 Title: Supplementary Figure 1. Description: Manhattan plot for results obtained from LEAP for the GWAS with the BMI phenotype. The y-axis shows -log10 of the p-values from the test for association between BMI extremes and genotype and the x-axis shows genomic position of the SNP. The blue line indicates the standard threshold for a suggestive association (p-value <1×10^−5^). The red line indicates the standard threshold for a genome-wide significant association (p-value <5×10^−8^). [file 12864_2022_8297_MOESM1_ESM.png]

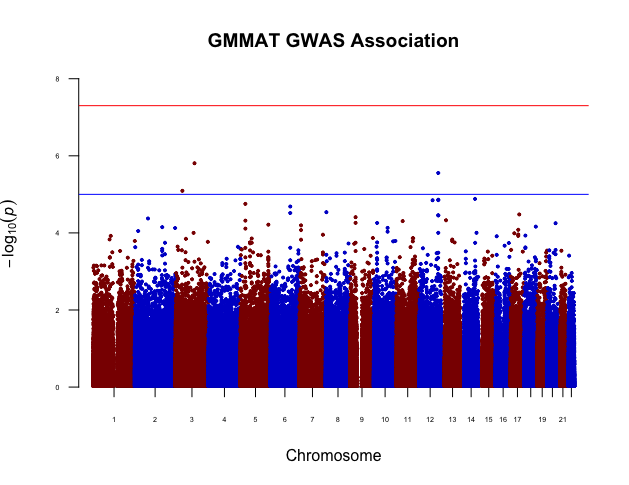

Supplement: Supplementary file 2 — Additional file 2 Title: Supplementary Figure 2. Description: Manhattan plot for results obtained from GMMAT for the GWAS with the BMI phenotype. The y-axis shows -log10 of the p-values from test for association between BMI extremes and genotype and the x-axis shows genomic position of the SNP. The blue line indicates the standard threshold for a suggestive association (p-value <1×10^−5^). The red line indicates the standard threshold for a genome-wide significant association (p-value <5×10^−8^). [file 12864_2022_8297_MOESM2_ESM.png]

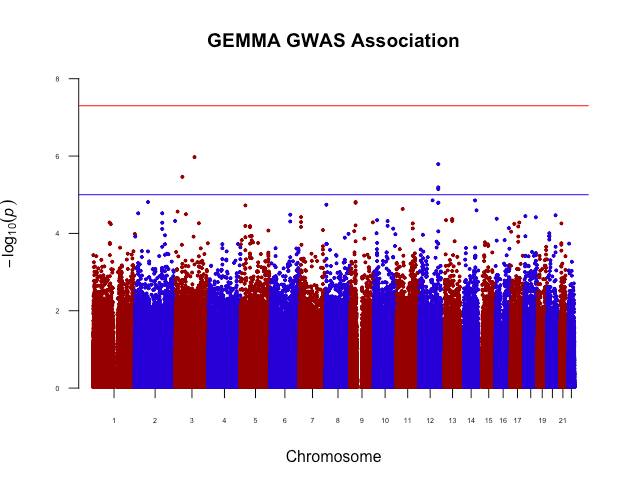

Supplement: Supplementary file 3 — Additional file 3 Title: Supplementary Figure 3. Description: Manhattan plot for results obtained from GEMMA for the GWAS with the BMI phenotype. The y-axis shows -log10 of the p-values from test for association between BMI extremes and genotype and the x-axis shows genomic position of the SNP. The blue line indicates the standard threshold for a suggestive association (p-value <1×10^−5^). The red line indicates the standard threshold for a genome-wide significant association (p-value <5×10^−8^). [file 12864_2022_8297_MOESM3_ESM.png]

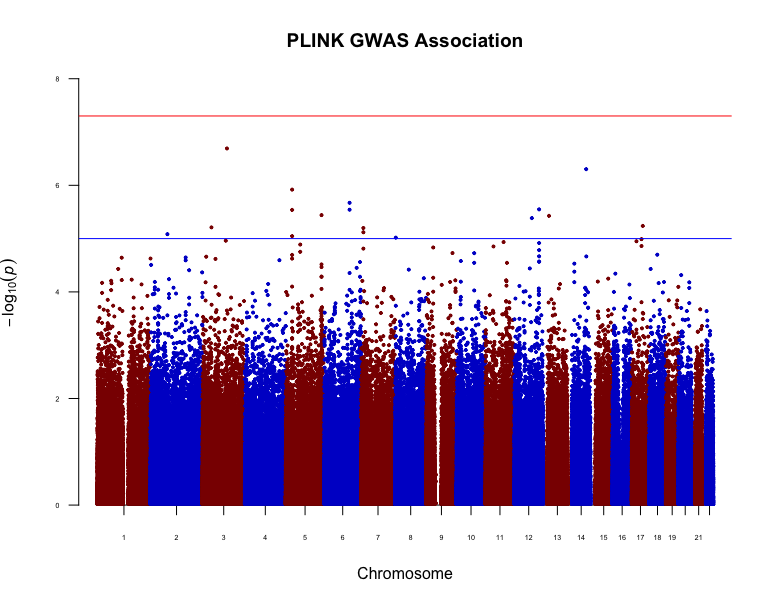

Supplement: Supplementary file 4 — Additional file 4 Title: Supplementary Figure 4. Description: Manhattan plot for results obtained from PLINK (uncorrected logistic regression) for the GWAS with the BMI phenotype. The y-axis shows -log10 of the p-values from test for association between BMI extremes and genotype and the x-axis shows genomic position of the SNP. The blue line indicates the standard threshold for a suggestive association (p-value <1×10^−5^). The red line indicates the standard threshold for a genome-wide significant association (p-value <5×10^−8^). [file 12864_2022_8297_MOESM4_ESM.png]
